# Genome-Wide Identification of *TLP* Gene Family in *Populus trichocarpa* and Functional Characterization of *PtTLP6*, Preferentially Expressed in Phloem

**DOI:** 10.3390/ijms25115990

**Published:** 2024-05-30

**Authors:** Mengjie Guo, Xujun Ma, Shiying Xu, Jiyao Cheng, Wenjing Xu, Nabil Ibrahim Elsheery, Yuxiang Cheng

**Affiliations:** 1 State Key Laboratory of Tree Genetics and Breeding, Northeast Forestry University, Harbin 150040, China; mengjieguo327@163.com (M.G.); maxj@nefu.edu.cn (X.M.); 13178123918@163.com (S.X.); chengjiyao1993@163.com (J.C.); 2 College of Life Sciences, Northeast Forestry University, Harbin 150040, China; xuwenjinglucky@163.com; 3 Agricultural Botany Department, Faculty of Agriculture, Tanta University, Tanta 31527, Egypt; nshery@yahoo.com

**Keywords:** *TLP*, *Populus trichocarpa*, peroxisome, reactive oxygen species, early flowering

## Abstract

Thaumatin-like proteins (TLPs) in plants are involved in diverse biotic and abiotic stresses, including antifungal activity, low temperature, drought, and high salinity. However, the roles of the *TLP* genes are rarely reported in early flowering. Here, the *TLP* gene family was identified in *P. trichocarpa*. The 49 *PtTLP* genes were classified into 10 clusters, and gene structures, conserved motifs, and expression patterns were analyzed in these *PtTLP* genes. Among 49 *PtTLP* genes, the *PtTLP6* transcription level is preferentially high in stems, and GUS staining signals were mainly detected in the phloem tissues of the *PtTLP6*pro::GUS transgenic poplars. We generated transgenic *Arabidopsis* plants overexpressing the *PtTLP6* gene, and its overexpression lines showed early flowering phenotypes. However, the expression levels of main flowering regulating genes were not significantly altered in these *PtTLP6*-overexpressing plants. Our data further showed that overexpression of the *PtTLP6* gene led to a reactive oxygen species (ROS) burst in *Arabidopsis*, which might advance the development process of transgenic plants. In addition, subcellular localization of *PtTLP6*-fused green fluorescent protein (GFP) was in peroxisome, as suggested by tobacco leaf transient transformation. Overall, this work provides a comprehensive analysis of the *TLP* gene family in *Populus* and an insight into the role of TLPs in woody plants.

## 1. Introduction

Thaumatin-like proteins (TLPs) belong to the PR-5 family of pathogenesis-related proteins (PRs), which were named based on the high sequence similarity to the sweet-tasting thaumatin protein from *Thaumatococcus daniellii Benth* [1] {XE “[1]”}{XE “[1]”}{XE “[1]”}. Members of the PR-5 family exhibit various biological functions such as peptide binding activity, β-1,3-glucanase activity, antifungal properties, osmotic regulation, and enzyme inhibition [2]. Most TLPs contain a highly conserved motif, which is G-X-[GF]-X-C-X-T-[GA]-D-C-X-(1,2)-G-X-(2,3) –C and a REDDD (arginine, glutamic acid, and three aspartic acid residues) structure, of which ten or sixteen conserved cysteine residues can form five or eight disulfide bonds [3,4,5]. These disulfide bonds help to maintain the 3D structure of TLPs under unfavorable conditions of high temperature or low pH, and it is essential for the antifungal activity of TLPs [6].

*TLP* genes have been reported in fungi, animals, and plants. The *TLP* genes in fungi, including in *Irpex lacteus*, *Lentinula edodes*, and *Rhizoctonia solani*, were first reported by Grenier et al. [7]. After searching for homologs in the genomic databases at NCBI using the amino acid sequences of reported TLPs, it has been revealed that the *TLP* gene family generally contains 2–3 members in fungi [2]. The *TLP* genes of animals were first found in *Caenorhabditis elegans*, and according to its sequence, *TLP* homologs have also been found in other insects, including *Schistocerca gregaria*, *Aphididae,* and *Pyrochroidae* [3,8]. *TLP* genes are ubiquitous in plants, including angiosperms, gymnosperms, and bryophytes. The 44, 49, and 24 TLPs were identified in the databases of rice, maize, and *Arabidopsis* [9]. Although there are many members of the *TLP* gene family in plants, their functions are relatively obscure. In particular, the roles of *TLP* genes remain largely unknown in *Populus*.

*TLP* genes have been reported to respond to different kinds of biotic stresses in plants. Cold-induced *TLP* genes in winter wheat epidermis displays antifungal activity against snow mold [6,10], while *VvTLP-1* from grapes significantly inhibits the in vitro spore germination and hyphal growth of *Botrytis cinerea* [11,12]. Overexpression of *ObTLP1* in *Arabidopsis* enhances transgenic plant resistance to *Sclerotinia sclerotiorum* and *Botrytis cinerea* [13], and *AsPR5* plays the role in garlic and *Arabidopsis* resistance to gray mold [14]. Overexpression of *GbTLP1* in cotton fibers affects secondary cell wall development and leads to enhancing the resistance of transgenic lines to *Verticillium dahliae* [15]. The *VqTLP29* gene in grapes plays a role in the closure of stomatal immunity in response to pathogen-associated molecular patterns, increasing resistance to powdery mildew [12]. *TLP* genes also play the important roles in abiotic stress, including low temperature, drought, and high salinity. Overexpression of the *AnTLP13* gene enhances the tolerance of tobacco to cold stress [5], and overexpression of *ObTLP1* in *Arabidopsis* and of *VqTLP*29 in grapes enhances resistance to methyl jasmonate, salicylic acid, and ethylene [12,13]. In addition, some TLPs participate in other processes such as floral organ formation, seed germination, and secondary wall development in cotton fibers [15,16,17].

Currently, the structures of several TLPs, including thaumatin, zeamatin, tobacco PR-5, and osmotin, have been identified [18,19,20,21]. All have similar three-dimensional structure, generally including three regions, with an acid/basic separation structure between regions I and II to bind different protein receptors [22]. In some plant TLPs with known antifungal activities, the cleft is acidic and contains five highly conserved amino acids (arginine, glutamate, and three aspartate residues). Identification of the *P. trichocarpa TLP* gene family can reveal the characteristics, evolutionary relationships, and structural information of plant *TLP* genes. In this study, we identified the *TLP* genes in *P. trichocarpa* and explored the function of phloem-preferentially-expressed *PtTLP6* through its overexpression in *Arabidopsis*.

## 2. Results

### 2.1. Identification and Phylogenetic Analysis of TLP Gene Family in P. trichocarpa

To identify the *TLP* gene family in *P. trichocarpa*, we searched the Pfam thaumatin domain (PF00314) and the reported *Arabidopsis* TLPs sequences as the queries in its genome. As a result, a total of 49 *TLP* genes were identified in the *P. trichocarpa* genome. All *TLP* genes were named as *PtTLP*1 to *PtTLP*49 according to the successive order of genes in the chromosomes, and the length of proteins encoded by the *PtTLP* genes varied from 155 to 686 amino acids (Table S1). More information on the *PtTLP* genes, including molecular weight, theoretical pI, instability, aliphatic, grand average of hydropathicity, and the proposed protein subcellular localization, are shown in Table S2.

**Figure 1:**
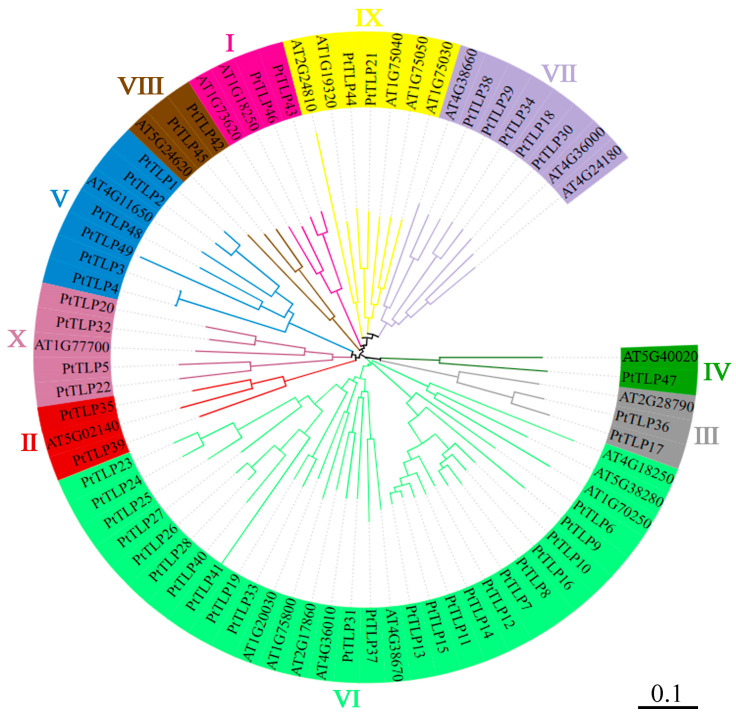
Phylogenetic analysis of *Populus* and *Arabidopsis TLP* genes. A total of 49 *PtTLP* genes and 24 *AtTLP* genes were aligned with Clustal W, and the phylogenic tree was constructed by MEGA 11 using the neighbor-joining method with 1000 bootstrap replication. All *TLP* genes were classified into ten clusters with different colors. The branch lengths of the phylogenetic tree represent genetic distance; the scale represents 0.1.

We constructed a phylogenetic tree with the 49 *PtTLP* genes, as well as 24 known *Arabidopsis TLP* genes (Figure 1). According to the phylogeny of *Arabidopsis TLP* genes and *Oryza TLP* genes [23], 49 *PtTLP* genes were classified into 10 clusters, named as Cluster I-X. The phylogenetic analysis showed that each cluster contained at least one *AtTLP* gene from *Arabidopsis*, suggesting a homologous relationship between *PtTLP* genes and *AtTLP* genes. Among ten clusters, cluster Ⅵ contains the most *PtTLP* members, with 23 *PtTLP* genes, while cluster Ⅳ contains the fewest members, with only one *PtTLP* gene.

### 2.2. Gene Structures, Conserved Motifs, and Expression Patterns in PtTLP Genes

We mapped 49 *PtTLP* genes on chromosomes by TBtools-Ⅱ (Figure S1). The physical locations of these *PtTLP* genes on chromosomes were scattered and uneven on 14 chromosomes. Chromosome 1 contained the maximum number (17 members) of *PtTLP* genes, while chromosomes 10 and 17 contained only one member. Tandem and segmental duplications are the main mechanisms leading to gene family expansion [24,25,26]. Based on the searches in the PGDD database [27], the *PtTLP* gene family contained 17 segmental duplicate gene pairs, which are *PtTLP5/22*, *PtTLP21/44*, *PtTLP23*/*40*, *PtTLP20/32*, *PtTLP17/36*, *PtTLP42/45*, *PtTLP43/46*, *PtTLP18/29*, *PtTLP18/30*, *PtTLP18/33*, *PtTLP18/37*, *PtTLP29/30*, *PtTLP29/33*, *PtTLP29/37*, *PtTLP30/33*, *PtTLP30/37*, and *PtTLP33/37* (Figure S1). Among these gene pairs, *PtTLP18*, *29*, *30*, *33*, and *37,* located on the replication blocks, formed a reciprocal duplicate gene group. The replication blocks are presumed to have arisen from the salicoid-specific genome duplication, and the specific locations on the chromosomes have been provided by Tuskan GA et al. [28]. We carried out a sequence alignment of the reciprocal duplicate genes, and their sequences exhibited high similarities (Figure S2). Seven groups of *PtTLP* genes, namely *PtTLP2/3/4*, *PtTLP18/19*, *PtTLP30/31*, *PtTLP33/34*, *PtTLP37/38*, *PtTLP40/41*, and *PtTLP48/49*, were located in tandem on chromosomes 1, 2, 5, 5, 9, 11, and 18, respectively. *PtTLP2/3/4*, *PtTLP40/41*, and *PtTLP48/49* belonged to the same cluster in the phylogenic tree (Figure 1). This suggests that they might arise from recent tandem duplication events [29]. Some tandem genes like *PtTLP18/19* and *PtTLP30/31* were grouped into different gene clusters, possibly because they have undergone neofunctionalization [28]. The indicator for this process is the ratio of nonsynonymous substitution rate (Ka) to synonymous substitution rate (Ks) between gene pairs being less than 1 [25]. We obtained the Ka and Ks of *PtTLP* tandem genes from PGDD [27], and the Ka/Ks ratios of *PtTLP18/19*, *PtTLP30/31*, and *PtTLP33/34* are less than 1. In addition, the sequence identity and similarity of tandem gene pairs were shown in Figure S3; for instance, those of *PtTLP18/19* were 52.74% and 63.69%. Overall, these results suggest that both tandem and segmental duplications play an important role in the expansion of the *PtTLP* gene family.

**Figure 2:**
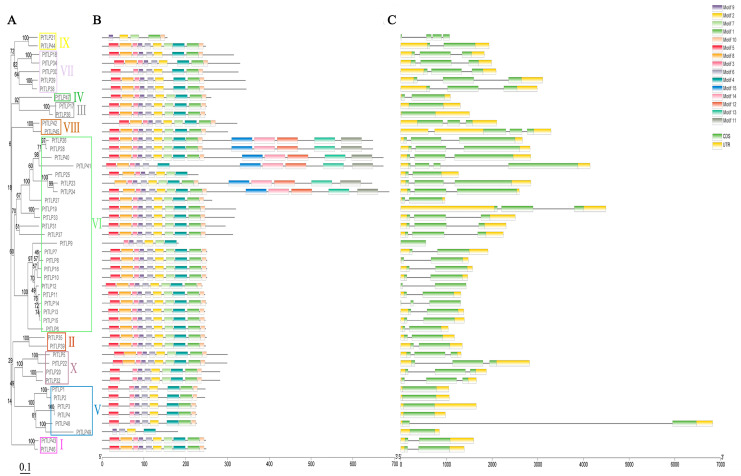
Phylogenetic tree, protein motif, and gene structure of 49 *PtTLPs* genes. (**A**) Phylogenetic tree. Branch lengths represent genetic distances, and the scale bar represents 0.1. We labeled cluster I–X and marked them with boxes of different colors. (**B**) Protein motifs. Conserved motifs 1–15 are represented by colored boxes, while non-conserved sequences are indicated by gray lines. (**C**) Gene structure. Yellow boxes represent exons, black lines represent introns, and untranslated regions (UTRs) are showed by green boxes. The sizes of the exons and introns can be estimated by the scale at the bottom.

Furthermore, we analyzed the exon/intron arrangements of the 49 *PtTLP* genes based on their phylogenetic tree (Figure 2A). Most *PtTLP* genes within the same category exhibited similar gene structures in terms of exon/intron numbers and lengths, while those in different categories displayed distinct exon/intron structural features (Figure 2C). Genes in categories VII, VIII, and X all have 3 exons. In clusters I, II, and IV, all genes have two exons, whereas in clusters III and V, most genes only have one exon, except *PtTLP48,* with two exons. Clusters VI and IX show greater variations in gene structure, with exon numbers ranging from 1 to 4, but the number of the exon was similar within each subgroup. We analyzed the distribution of conserved motifs and captured 15 motifs using the MEME tool (Figure 2B and Figure S4). The length of the motifs of *PtTLP* genes ranged from 11 to 50 amino acids, and the number of conserved motifs varied from 5 to 15 in each of the *PtTLP* genes. Motifs 1 to 10 appeared in almost all members of *PtTLP* genes, whereas motifs 11 to 15 were specific to cluster Ⅰ.

**Figure 3:**
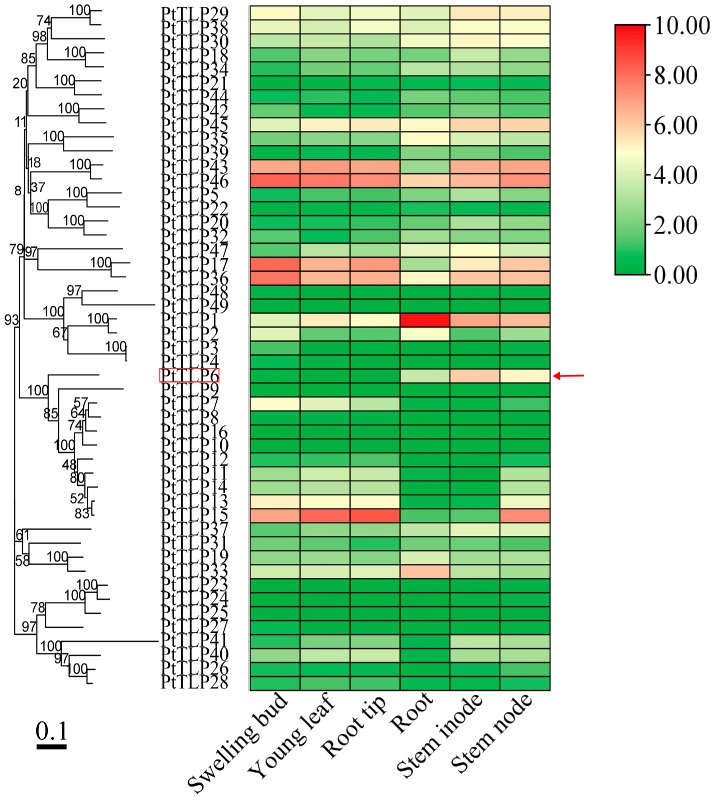
Hierarchical clustering of expression profiles of *PtTLP* genes in different tissues. The microarray data were downloaded from the JGI Data Portal (https://phytozome-next.jgi.doe.gov/geneatlas/; accessed on 10 October 2023) [30]. The color scale at the right of the graph indicates log2 expression values. PtTLP6 was marked with a red box and a red arrow. Branch lengths of the phylogenetic tree represent genetic distances, and the scale bar represents 0.1.

To examine the expression patterns of the *PtTLP* genes, we analyzed their transcription levels across multiple tissues and organs (including swelling bud, young leaf, root tip, root, stem inode, and stem node) using the Gene Atlas dataset downloaded from the JGI Data Portal (Table S3). The heat map exhibited that the majority of *PtTLP* genes had tissue-specific or preferential expression patterns (Figure 3). *PtTLP1* and *PtTLP33* were mainly expressed in roots, but the transcription levels of *PtTLP13*, *PtTLP17*, *PtTLP36*, *PtTLP43*, and *PtTLP46* were lower in root and higher in swelling bud. Notably, *PtTLP6* showed lower expression levels in swelling bud, young leaf, root tips, and roots, but significantly higher expression specifically in stem node and inode. These results imply that the *PtTLP* genes may be involved in various processes of *P. trichocarpa* growth and development.

### 2.3. PtTLP6 Highly and Preferentially Expressed in Populus Phloem

**Figure 4:**
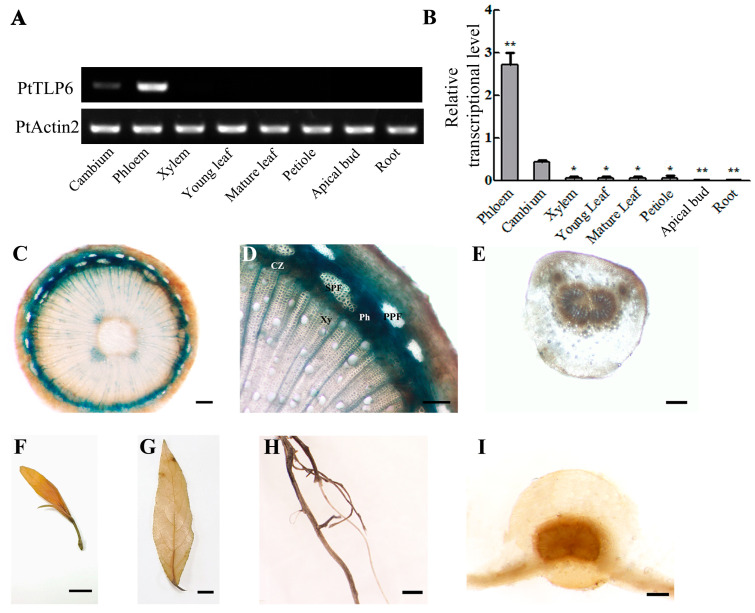
*PtTLP6* gene expression pattern in various tissues of *P. trichocarpa*. (**A**) RT-PCR analysis of *PtTLP6* gene expression in different tissues, including xylem, cambium, phloem, young leaf, mature leaf, petiole, apical bud, and root. (**B**) RT-qPCR analysis of *PtTLP6* gene expression in different tissues. The expression level of *PtActin2* was used as an internal control. Statistically significant differences between cambium and other tissues were determined by *t*-test. (**C**–**I**) GUS staining of different tissues in transgenic *PtTLP6*pro::GUS plants. (**C**,**D**) Cross-section of stem internode 9. (**E**,**I**) cross-sections of the 5th leaf petiole and main vein. (**F**–**H**) Apical bud, mature leaf, and root. CZ, cambium zone; PPF, primary phloem fiber; SPF, secondary phloem fiber; Ph, phloem; Xy, xylem. In (**B**), the error bars represent SDs (*n* = 3). *p* < 0.05 was marked as *, *p* < 0.01 was marked as **. Scale bars in (**C**–**E**,**I**) represent 200 μm; in (**F**–**H**), 1 cm.

To understand the function of the *PtTLP6*, we examined its expression pattern in various tissues in detail. Among xylem, cambium, phloem, young leaf, mature leaf, petiole, apical bud, and root tissues, RT-PCR data showed the highest expression level of *PtTLP6* in phloem (Figure 4A). RT-qPCR analysis further revealed expression levels of *PtTLP6* in different tissues (Figure 4B), which was in agreement with the results of RT-PCR analysis. These data suggested that the *PtTLP6* was likely related to stem development or phloem function.

Next, we isolated the *PtTLP6* gene promoter with the length of 2.38 kb, constructed the *PtTLP6*pro::GUS binary vector, and obtained transgenic *P. trichocarpa* plants expressing a GUS gene driven by *PtTLP6* promoter. In 3-month-old *PtTLP6*pro::GUS transgenic plants, GUS staining signals were intensively detected in secondary phloem fibers, phloem parenchyma cells, sieve tubes, and companion cells in the tested stem internode 9 (Figure 4C,D), which transport the sugars produced by photosynthesis from the leaves to other parts of the tree for growth and energy. Similar results were observed in other stem internodes including the internodes 10 to 20. The cambium zone, xylem ray cells, and primary phloem fibers of stem internode 9 also showed slight GUS signals. Xylem and phloem cells are usually produced by inward and outward division of vascular cambium cells, respectively [31]. It is thus reasonable that *PtTLP6* remains at a low expression level in the cambium. No GUS signal was observed in the cross section of the petiole and main vein of the 5th leaf, apical bud, mature leaf, and root (Figure 4E–I). Our findings suggest that *PtTLP6* is highly and preferentially expressed in phloem tissues of poplars.

### 2.4. Overexpression of PtTLP6 Gene in Arabidopsis Leads to Early Flowering

**Figure 5:**
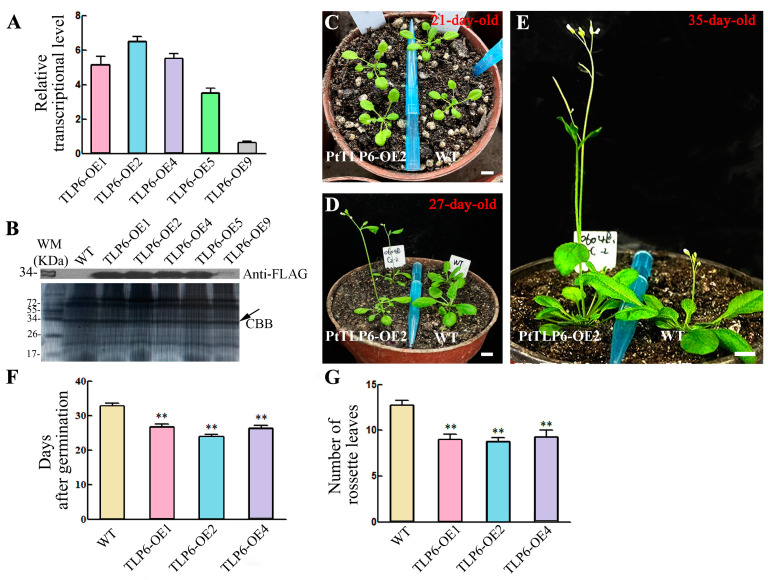
Overexpression of *PtTLP6* gene in *Arabidopsis* results in early flowering. (**A**) RT-qPCR analysis of *PtTLP6* gene expression in transgenic *Arabidopsis* plants. *PtActin2* was used as an internal control. (**B**) Western blot analysis of PtTLP6 protein levels in transgenic *Arabidopsis* plants. Total proteins were extracted from the leaves of WT, *PtTLP-OE*1, 2, 4, 5, and 9 transgenic lines, separated on a 12% SDS-PAGE gel, and immunoblotted with anti-FLAG antibody (Abcam, Cambridge, UK). A replicate coomassie brilliant blue (CBB)-stained gel is shown to confirm equal loading. The black arrow shows the approximate position of the PtTLP6 protein, which is 30 kDa in size. (**C**–**E**) Photos were taken from WT and *PtTLP6-OE*2 plants grown on soils for 21 d (**C**), 27 d (**D**), and 35 d (**E**), respectively. In view of similar phenotypes of *PtTLP6-OE*1, 2, and 4 plants, only *PtTLP6-OE*2 is shown here. (**F**) Statistics of WT and *PtTLP6-OE* plant flowering time. (**G**) Statistics of the number of rosette leaves from WT and *PtTLP6-OE* plants when flowering. Data are means ± standard error of three technical replicate results. Scale bars, 1 cm; *p* < 0.01, **.

To gain insight into the function of *PtTLP6* gene, we generated transgenic *Arabidopsis* lines that overexpressed the *PtTLP6* (fused with the FLAG tag at its carboxyl terminus), called *PtTLP6-OE*. We performed RT-qPCR analysis of *PtTLP6* in five transgenic lines using the *AtActin2* gene as an internal reference. *PtTLP6-OE*1, 2, and 4 exhibited high transcription levels of *PtTLP6*, which were 5–7 times higher than those of *AtActin2* (Figure 5A). Western blot analysis using the anti-FLAG antibody (Abcam, Cambridge, UK) showed that four of five transgenic lines, *PtTLP6-OE*1, 2, 4, and 5, had significantly high PtTLP6 protein levels (Figure 5B). This suggests that these lines overexpressed the *PtTLP6* in *Arabidopsis*.

Next, we planted *PtTLP6-OE*1, 2, 4, and wild type (WT) in the greenhouse to observe their phenotypes. It showed that the phenotype of the overexpression lines was similar to that of the WT at the seedling stage (Figure S5). At 21 days, there was no difference in the phenotypes, the number of rosette leaves, and the leaf size, between the overexpression lines and the WT (Figure 5C). The *PtTLP6-OE* lines flowered at 27 days, while the WT had not bolted yet (Figure 5D). By 35 days, the WT had bolted, while the overexpression lines had already developed the siliques (Figure 5E). We counted the days after germination to flowering and the number of rosette leaves of flowering *PtTLP6-OE* lines and WT plants (Figure 5F,G). After approximately 32 days of growth on soil, WT plants flowered, while *PtTLP6-OE* lines did at 25–27 days. When flowering, there were 12–13 rosettes in WT and 8-10 in *PtTLP6-OE* lines. The findings indicate that overexpression of *PtTLP6* gene in *Arabidopsis* leads to early flowering, possibly by accelerating the growth process of transgenic plants.

### 2.5. Expression Levels of Flowering Regulating Genes Were Not Altered in PtTLP6-OE Arabidopsis

**Figure 6:**
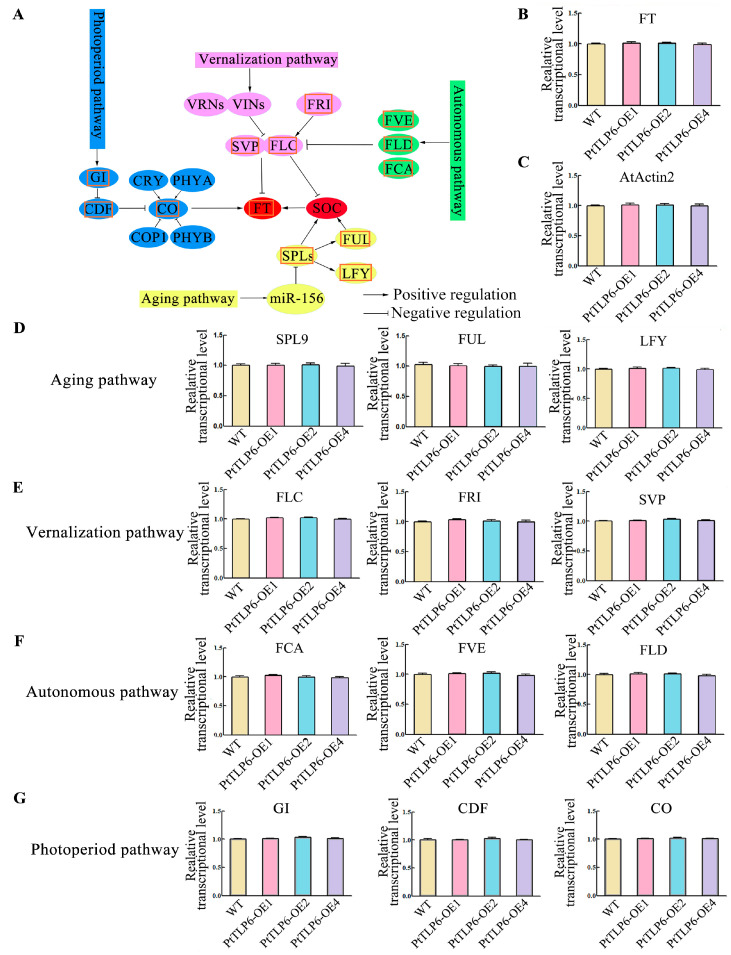
Transcription levels of main flowering pathway genes in WT and *PtTLP6-OE* lines. (**A**) Four major pathways regulating flowering time and the marker genes involved. The marker genes labelled with red boxes were selected for RT-qPCR analysis. (**B**) Relative expression level of FT in WT and three *PtTLP6-OE* lines. (**C**) Relative expression level of the housekeeping gene *AtActin2* of WT and three *PtTLP6-OE* lines. (**D**–**G**) Relative expression levels of marker genes of aging pathway, vernalization pathway, autonomous pathway and photoperiod pathway of WT and three *PtTLP6-OE* lines. Three biological experiment replicates and three technical experiment replicates were performed for each outcome. Data are means ± SD (*n* = 3). *FT*, FLOWERING LOCUS T; *SPL9*, SQUAMOSA PROMOTER BINDING PROTEIN-LIKE 9; *FUL*, FRUITFULL; *LFY*, LEAFY; *FLC*, FLOWERING LOCUS C; *SVP*, SHORT VEGETATIVE PHASE; *FRI*, FLOWERING LOCUS A; *FVE*, MULTICOPY SUPPRESSOR OF IRA1 4; *FLD*, FLOWERING LOCUS D; *FCA*, FLOWERING CONTROL LOCUS A; *GI*, GIGANTEA; *CDF,* CYCLING DOF FACTOR 5; *CO*, CONSTANS.

To examine whether early flowering results from the expression changes of flowering marker genes, we collected the sixth rosette leaves of WT and *PtTLP6-OE* lines to conduct RT-qPCR analysis of FLOWERING LOCUS T (*FT*), a key gene regulating the flowering time and flowering regulatory genes in various pathways [32,33]. The results showed that the transcription levels of *FT* were nearly unchanged between the WT and *PtTLP6-OE* lines (Figure 6B). In the aging pathway, the expression levels of *SPL9*, *FUL,* and *LFY* showed no significant variation among WT and *PtTLP6-OE* lines (Figure 6D). In the vernalization pathway, the transcription levels of the *FLC*, *SVP,* and *FRI* were not significantly altered among the WT and *PtTLP6-OE* lines (Figure 6E). The expression levels of *FVE*, *FLD,* and *FCA* in the autonomous pathway were not significantly changed among the WT and *PtTLP6-OE* lines (Figure 6F). Similarly, the transcription levels of *GI*, *CDF*, and *CO* from the photoperiod pathway almost stayed in line among the WT and *PtTLP6-OE* lines (Figure 6G). Overall, the transcription levels of these flowering regulatory genes tested remained largely unchanged between the WT and *PtTLP6-OE* transgenic lines.

### 2.6. Overexpression of PtTLP6 Induces ROS Burst in Arabidopsis

**Figure 7:**
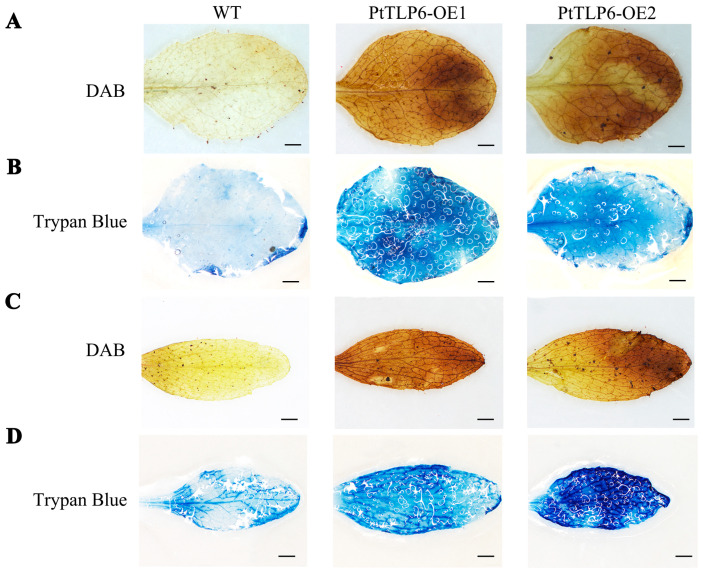
Cell viability staining of the rosette and cauline leaves of WT and *PtTLP6-OE* lines. (**A**,**C**) DAB staining of the rosette and cauline leaves from WT and *PtTLP6-OE* lines. (**B**,**D**) Trypan blue staining of the rosette and cauline leaves from WT and *PtTLP6-OE* lines. The scale bars are 100 µm.

In view of the advanced growth and development of *PtTLP6-OE* lines in comparison to the WT, we examined the ROS levels in these transgenic plants using DAB staining analysis. Both rosette and cauline leaves of the *PtTLP6-OE* lines showed obviously darker color than those of the WT (Figure 7A,C), indicating that the amount of hydrogen peroxide, a major ROS, was burst in *PtTLP6-OE* lines. Next, trypan blue staining was performed for assaying the proportion of dead cells in the rosette and cauline leaves of the WT and *PtTLP6-OE* lines. The data showed that compared with the WT, *PtTLP6-OE* lines had a much higher proportion of dead cells in the leaves (Figure 7B,D), revealing that excessively accumulated ROS might accelerate programmed cell death (PCD) in transgenic plants.

### 2.7. PtTLP6 Is Localized in Peroxisomes

**Figure 8:**
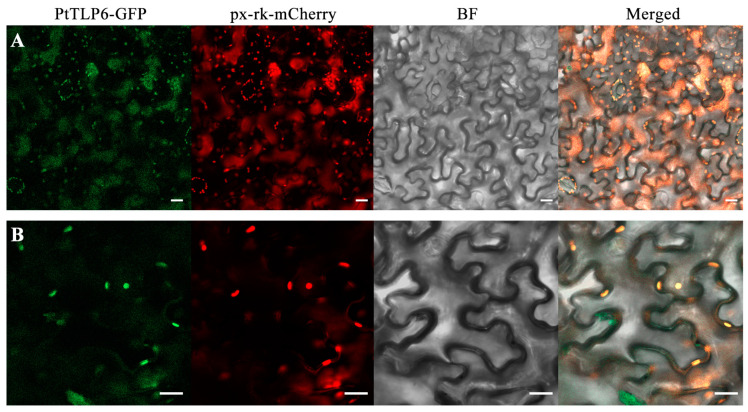
PtTLP6-GFP protein is localized in peroxisomes. (**A**,**B**) Fluorescence images were taken from tobacco leaf mesophyll cells under different channels of laser confocal microscopy at low (**A**) and high (**B**) magnifications. The GFP channel is used for the localization of PtTLP6-GFP protein and mCherry channel for the localization of px-rk-mCherry in peroxisomes. BF, bright field images. Merged, imaging of all channels. Bars, 20 μm.

To further understand the role of *PtTLP6* in *PtTLP6-OE Arabidopsis* plants, we determined subcellular localization of *PtTLP6* in tobacco leaf mesophyll cells. The CDS of *PtTLP6* gene was fused into plant expression vector pGWB_5_, which contains a green fluorescent protein (GFP) gene. The resultant vector with the *PtTLP6-GFP* gene, the mt-rk-mCherry control vector containing the first 29 aa of yeast cytochrome c oxidase IV (*ScCOX4*) for mitochondria localization, and the px-rk-mCherry control vector containing peroxisomal targeting signal 1 (*PTS1*) for peroxisome localization were transformed into *Agrobacterium tumefaciens* [34,35,36] and co-transfected into the leaves of one-month-old tobacco. After one day of culture in darkness, tobacco mesophyll cells were observed under a laser confocal microscope. As a result, the fluorescence signal of PtTLP6-GFP protein completely co-localized with that of a peroxisome protein marker px-rk-mCherry (Figure 8). As a negative control, the fluorescence signal of a mitochondrial protein marker mt-rk-mCherry did not co-localize with that of PtTLP6-GFP (Figure S6). These findings reveal the localization of the PtTLP6-GFP protein in the peroxisomes of plant cells, which are the major organelle of cellular ROS production [37].

## 3. Discussion

In this study, we identified 49 *PtTLP* genes in *P. trichocarpa*, but a previous study presented 50 *PtTLP* genes [38]. One reason is that the database used here is the latest *P. trichocarpa* v4.1 of the Gene Atlas Project, from which many duplicate genes were deleted. In genome evolution, a gene generates two or more copies through gene duplication, which expands gene family. In the study of Liu et al. [2], 28 TLPs were shown in Arabidopsis by BLAST from genomic databases at NCBI. In fact, using the 28 TLPs as a query in the Arabidopsis Information Resource (TAIR 10), only 24 TLPs existed in the Arabidopsis genome. So, the 24 AtTLPs were included in the phylogenetic analysis of this study, which is consistent with other studies [9]. The number of *PtTLP* members was 49, far more than that of *AtTLP* members, perhaps because *PtTLP* genes has undergone more rounds of gene duplication. (Figure S1). Based on the phylogenetic classification of *Arabidopsis TLP* genes and *Oryza TLP* genes [23], 49 *PtTLP* genes were grouped into 10 clusters. In the study of Ren et al. [39], the seven clusters of wheat *TLP* genes were shown in the phylogenetic tree, but each did not require at least one *AtTLP*. In our study, each cluster of the PtTLP genes in the phylogenetic tree contains at least one *AtTLP* member, consistent with earlier studies [23,40]. *PtTLP* genes exhibit some conserved motifs, but specific motifs have evolved in different clusters, suggesting that *PtTLP* genes within the same cluster may share functional similarities.

Previous studies on *TLP* genes have focused on their functions in pathogenesis resistance. In this study, overexpression of the *PtTLP6* gene led to early flowering of transgenic *Arabidopsis* plants. Under the same growth conditions, the OE-*PtTLP6 Arabidopsis* plants flower approximately 8 days earlier than the WT plants (Figure 5E). With the evolution of plants, complex flowering regulatory pathways have been formed through the interaction and mutual influence of the plant itself and external environmental factors. In *Arabidopsis*, studies have shown that a total of 306 genes are involved in regulating the process of flowering transition [41]. These genes have been divided into eight pathways: the photoperiod pathway, vernalization pathway, aging pathway, hormones pathway, temperature pathway, autonomous pathway, circadian clock pathway, and sugar pathway [32,33]. In our study, to seek the reasons for the early flowering in *PtTLP6-OE* lines, we analyzed the transcription levels in marker genes of the photoperiod pathway, autonomous pathway, vernalization pathway, and aging pathway in both the WT and *PtTLP6-OE* lines. The transcription levels of these genes were the same as those of the WT and *PtTLP6-OE* lines. Certainly, it could not be ruled out whether the expression of other flowering regulatory genes was altered in the *PtTLP6-OE* lines.

Our findings have revealed that *PtTLP6* is specially expressed in the phloem (Figure 4). In plants, the main function of phloem is to transport photosynthetic products and a variety of signal substances to promote plant growth [42]. For example, *CLE45* confers high-temperature tolerance during reproduction through its long-distance phloem transport [43]. Proteins are also important long-distance signals in the phloem vascular bundles, with a prime example being the *FT* protein. *FT* can be transported from leaves to top buds to regulate flowering time [44]. Other genes such as *MRF1* have been reported to localize in the phloem and regulate flowering. *Arabidopsis* mrf1 mutants have shown the delayed flowering phenotype and, conversely, *MRF1*-overexpressing plants exhibited early flowering [45]. It has been reported that the maturity of the phloem affects the transportation of water and nutrients in plants, which could impact their growth and flowering [46]. The more mature the phloem is, the more effectively the plants are able to transport water and nutrients to the growing parts, which could promote plant growth and development.

It is possible that overexpression of *PtTLP6* accelerates maturation of the phloem tissues in transgenic plants. Some *TLP* genes play the roles in disease resistance because of their β-1,3-glucanase activity [2]. For instance, overexpression of *SlTLP5* or *SlTLP6* leads to resistance to soil-borne diseases in *Solanum lycopersicum* by enhancing β-1,3-glucanase activity [47]. It is known that β-1,3-glucanase can promote the deposition of secondary cell wall in plants [48]. Although we have not confirmed whether *PtTLP6* has the β-1,3-glucanase activity, overexpression of *PtTLP6* in Arabidopsis may enhance β-1,3-glucanase activity, potentially modifying the cell walls of the phloem and ultimately leading to premature maturation of the phloem. In this case, many signal substances promoting plant growth, including FT, could be early transported through vascular system of premature phloem, which eventually brings about early flowering of *PtTLP6-OE* plants.

In this study, DAB staining of rosette leaves and stem leaves has revealed that the ROS levels in OE-*PtTLP6* transgenic lines are higher than in WT plants (Figure 7A,C). A recent study has shown that overexpression of *TaTLP*1 in wheat regulates the activity of ROS-related enzymes and increases the expression of ROS burst-related genes [49]. It is well known that reactive oxygen species (ROS) can induce programmed cell death and apoptosis [50]. Maturation of the phloem vascular system is closely associated with the occurrence of programmed cell death [51]. Here, trypan blue staining has indicated that the leaves of OE-*PtTLP6* plants have a much higher proportion of dead cells than those of WT (Figure 7B,D). Thus, excessively accumulated ROS presumably accelerates programmed cell death in OE-*PtTLP6* plants, which advances prematurity of the vascular system in phloem.

In addition, our observations have suggested that overexpression of the *PtTLP6* gene accelerates the development process of transgenic *Arabidopsis* plants (Figure 5F,G). It has been reported that ROS are involved in the growth of a variety of floral organs at early developmental stages, not only in the apical part of the growing tissue [52]. Treatments of litchi trees with sodium nitroprusside (SNP), NO donor, or methyl vitiliginine dichloride (MV) have increased the contents of H_2_O_2_ and NO in mixed buds, and these ROS have promoted reproductive growth by inhibiting basic leaf growth [53,54]. Compared with the WT, the level of ROS is elevated in the leaves of *PtTLP6-OE* transgenic plants. The ROS burst is likely to generate signals in leaves that are associated with the induction of flowering or changes in leaf metabolism in preparation for the growth of reproductive structures.

In plant cells, ROS can be generated in mitochondria, chloroplasts, and peroxisomes [55]. Since the majority of the processes carried out in peroxisomes are oxidative metabolism, peroxisome may be a major site of intracellular H_2_O_2_ production. Our findings have demonstrated that *PtTLP6-GFP* is localized in the peroxisomes of tobacco leaf cells (Figure 8), providing a basis of mediating intracellular H_2_O_2_ production. According to previous studies, at least two different signals directing proteins to the peroxisomal matrix have already been identified [56]. Peroxisomal targeting signal (PTS) type 1 (*PTS1*) is an uncleaved tripeptide (serine-lysine-leucine or variants) at the C-terminal [57], while type 2 (*PTS2*) is an N-terminal cleavable peptide containing 11 to 36 amino acids [58]. However, *PtTLP6* does not contain these signal peptides, so further research is needed to determine how *PtTLP6* is targeted to the peroxisome.

In conclusion, we have identified 49 *TLP* genes in *P. trichocarpa* and analyzed their gene structures, conserved motifs, and expression patterns. Of all, *PtTLP6* is preferentially expressed in phloem, and the localization of *PtTLP6-GFP* is in peroxisome. Overexpression of *PtTLP6* in *Arabidopsis* leads to early flowering, as it mediates the ROS burst in transgenic plants, which might advance plant development process. Overall, this work uncovers a novel role of the *TLP* genes in plant growth and development.

## 4. Materials and Methods

### 4.1. Plant Material and Growth Conditions

Wild-type *P. trichocarpa* (Nisqually-1) and transgenic plants were planted in the greenhouse under the long day condition (16 h light/8 h dark) at 23–25 °C of the Northeast Forestry University, China. Both wild-type and transgenic plants were propagated in vitro on WPM (Lloyd & McCown Woody Plant Basal Medium w/Vitamins; PhytoTech Lab, L449) plates supplemented with 2.5% (*w/v*) sucrose. Transcriptional levels of genes in different tissues were measured using 4-month-old wild-type trees, including phloem, cambium, xylem, apical bud, young leaf, mature leaf, petiole, and root.

*Arabidopsis* (*Arabidopsis thaliana*; ecotype Columbia) plants were grown in a greenhouse (16 h of light/ 8 h of dark) with a light intensity of 80–120 μmol photons m^−2^ s^−1^ at 22 °C. The *Arabidopsis* seeds were germinated on sterilized 1/2MS (Murashige & Skoog Basal Salt Mixture; PhytoTech Lab, Lenexa, KS, USA) plates supplemented with 1% sucrose and the seedlings were transplanted into soil, which contained a 5:3:2 ratio of black soil, perlite, and vermiculite.

### 4.2. Identification of the TLP Genes in P. trichocarpa

We identified the *TLP* gene family in the genome of *P. trichocarpa* by the following two methods: (1) thaumatin domain (PF00314) as a search query in the whole genome; (2) amino acid sequences of 24 known *Arabidopsis TLP* genes as a search query in the Phytozome 13 database. The protein sequences, genomic sequences, and coding sequences (CDS) of all *TLP* genes were downloaded from *P. trichocarpa* v4.1 (https://phytozome-next.jgi.doe.gov/info/Ptrichocarpa_v4_1; accessed on 23 September 2023) [30]. All target proteins were scanned to detect the thaumatin domain (PF00314) with the SMART database [59]. The physicochemical properties of PtTLPs, such as theoretical pI and instability, were determined by TBtools-II software (version 2.096) [60]. The subcellular localization of *PtTLP* genes was predicted by TargetP v2.0 [61]. The phylogenetic tree was constructed using the neighbor-joining method of MEGA11 with a bootstrap value of 1000 replicates.

### 4.3. Chromosomal Duplication Analyses and Gene and Protein Structure Analysis

Chromosomal locations of 49 *PtTLP* genes were marked on the chromosome using TBtools-II software (version 2.096) [60]. Segmental and tandem duplications are the main mechanisms leading to gene family expansion [23]. Multiple genes undergo segmental duplication, followed by chromosomal rearrangements [24], which may occur in the specific regions of chromosomes which are more prone to recombination or other types of the chromosomal rearrangement events [23], which we called duplication blocks. The duplicated blocks were utilized to elucidate the expansion of the *PtTLP* gene family. Based on the previous study [28], the duplicated blocks were downloaded from the Plant Genome Duplication Database (PGDD) [27]. The definition of reciprocal duplicate genes is a group of genes that are each other’s duplicate genes and are located on the replication blocks provided by PGDD [27]. Tandem duplication in the genome was defined as those closely related genes falling within 50 kb of one another [62]. If these tandem duplicate genes were duplicated recently, we name them recent tandem duplication genes. They may exhibit higher sequence similarity and may belong to the same cluster in the phylogenic tree [29]. Conversely, if the duplication event occurred long ago, they may have accumulated more mutations over time. The *PtTLP* genes in tandem on chromosomes were screened and outlined by blue boxes in Figure S1. The sequence alignment of homologous genes was performed using MUSCLE (https://www.ebi.ac.uk/jdispatcher/msa/muscle; accessed on 1 April 2024) [63].

Gene structures, including the organization of exon and intron, and conserved motifs, were generated with TBtools-II software [60]. Conserved motifs of the *PtTLP* genes were analyzed by the Multiple Expectation Maximization for Motif Elucidation (MEME) system (https://meme-suite.org/meme/doc/meme.html; accessed on 30 September 2023) [64].

### 4.4. Microarray Data Analysis and Extraction of RNA, RT-PCR, and RT-qPCR

Tissue-specific expression data on the *PtTLP* genes were downloaded from the JGI Data Portal (https://phytozome-next.jgi.doe.gov/geneatlas/; accessed on 10 October 2023) [30]. The heat map was generated by TBtools-II software [60]. Total RNAs were isolated from all samples using pBIOZOL (Bio-Flux, Beijing, China) in accordance with the manufacturer’s instructions. All isolated RNA samples were assessed for purity, and the ratios of OD_260_/_280_ and OD_260/230_ were greater than 1.9 for RNA samples. Then the qualified RNA samples were used to synthesize the cDNAs by reverse transcription using the PrimeScript RT Reagent Kit with gDNA Eraser (TaKaRa, Dalian, China).

Expression of the *PtTLP6* gene in different tissues was examined by RT-PCR using 2 × Rapid Taq Master Mix (Vazyme, Nanjing, China). The reaction mixture contained 10 μL of Taq Master Mix, 1 μL of cDNA template, 0.5 μL of each gene-specific primer (10 μM) as well as 8 μL of distilled H_2_O. The PCR products were detected on the 2% agarose gels. Each reaction was performed three times to ensure the reproducibility of the results. RT-qPCR experiments were conducted using an ABI 7500 system (Applied Biosystems, Foster City, CA, USA) with SYBR Green (TaKaRa, Dalian, China). Each 20 µL reaction mixture contained 10 µL of 2 × TB Green Premix Ex Taq II (Tli RNaseH Plus), 1 µL of cDNA template, 0.4 µL of ROX Reference Dye II, 0.8 µL of each gene-specific primer, and 7 µL of distilled H_2_O. *PtActin2* was used as an internal control, and gene expression levels were calculated using the comparative cycle threshold (Ct, 2^−∆Ct^) method. Three biological experiment replicates and three technical experiment replicates were performed for each outcome. All primers used for RT-qPCR and RT-PCR are listed in Table S4.

### 4.5. Vector Construction

The genomic DNA was extracted from the leaves of 3-month-old WT using a plant genomic DNA extraction kit (Bioteke, Beijing, China). With genomic DNA and cDNAs as templates, promoter regions (2.38 kb) and CDS of *PtTLP6* were amplified by PCR and inserted into the vector pENTR/D-TOPO (Invitrogen, Carlsbad, CA, USA). After DNA sequencing, the *PtTLP6* promoter fragments and CDS from the entry clones were constructed into the binary vector pGWB_3_, pGWB_5_, and pGWB_11_ by the Gateway LR Clonase II enzyme (Invitrogen, Carlsbad, CA, USA) to generate *PtTLP6*pro::GUS, 35S::*PtTLP6-GFP* (green fluorescent protein), and 35S::*PtTLP6-FLAG* constructs. All target vectors were transformed into *Agrobacterium tumefaciens* strain GV3101 for future transformation. All primers are listed in Table S4.

### 4.6. Genetic Transformation

The transformation of *A. thaliana* was done by the floral-dip method [65] to generate *PtTLP6-OE* transgenic lines. The GV3101 strains with the pGWB_11_ vector containing 35S::*PtTLP6-FLAG* were incubated overnight in YEP liquid medium supplemented with 50 µg mL^−1^ kanamycin, gentamicin, and rifampicin at 28 °C. When the OD_600_ value of the cultures reached 1.0, cell pellet was collected by centrifugation at 22,000× *g* for 8 min and suspended in 250 mL transformation medium (containing 0.54 g of MS, 12.5 g of sucrose, 0.125 g of MES). The flowers of wild-type *Arabidopsis* were dipped for 5 min in the transformation medium and incubated in the dark for 24 h. Next, light culture was resumed, and seeds were collected after maturity. The positive transformants were selected on 1/2 MS plates containing 50 µg mL^−1^ of kanamycin, and several transgenic lines with high transcriptional levels of *PtTLP6* were further propagated.

For the *P. trichocarpa* transformation, the *A. tumefaciens* strains containing the *PtTLP6*pro::GUS vector were incubated overnight in 20 mL YEP liquid medium supplemented with 50 µg mL^−1^ kanamycin, gentamicin, and rifampicin at 200 rpm at 28 °C, until the OD_600_ value reached 0.8–1.0. Then, 2 mL of the bacterial cultures was inoculated into 50 mL YEP liquid medium supplemented with 50 µg mL^−1^ kanamycin, gentamicin, and rifampicin at 200 rpm at 28 °C, until the OD_600_ value reached 0.6. Cell pellet was collected by centrifugation at 2200× *g* for 10 min and suspended in 50 mL transformation medium (containing 0.04 g of WPM, 1.25 g of sucrose, 0.0136 g of MES). The wild-type stem explants were immersed in the transformation medium for 20 min with slight shaking, then incubated on WPM plates supplemented with 2.5% (*w/v*) sucrose in the dark for 48 h. Next, the stem explants were restored to light and cultured on WPM plates containing 2.5% (*w/v*) sucrose and 30 µg mL^−1^ kanamycin to screen the positive transgenic shoots. Detailed experimental procedures were carried out with reference to a previous study [66].

The transient transformation of tobacco mesophyll cells was performed in a previous study [67]; the *A. tumefaciens* strains with 35S::*PtTLP6-GFP* vector, mt-rk-mCherry control vector containing the first 29 aa of yeast cytochrome c oxidase IV (*ScCOX4*) for mitochondria localization, and px-rk-mCherry control vector containing peroxisomal targeting signal 1 (*PTS1*) for peroxisome localization [34,35,36] were all incubated overnight in 20 mL YEP liquid medium supplemented with 50 µg mL^−1^ kanamycin, gentamicin, and rifampicin at 200rpm at 28 °C, until the OD_600_ value reached 0.8. Then, 2 mL of the bacterial cultures was inoculated into 50 mL YEP liquid medium supplemented with 50 µg mL^−1^ kanamycin, gentamicin, and rifampicin at 200 rpm at 28 °C, until the OD_600_ value reached 0.5. Cell pellet was collected by centrifugation at 3000× *g* for 5 min and suspended in 50 mL transformation medium (containing 100 mM Mgcl_2_, 300 mM AS, 100 mM MES) for transient transformation. The transformation medium was then infiltrated into the air spaces of mesophyll cells of *Nicotiana benthamiana* leaves (also known as “agroinfiltration”), in which they transform plant cells, which in turn express the transgene within 48 h of dark culture.

### 4.7. GUS Staining

GUS staining analysis was performed using 3-month-old *PtTLP6*pro::GUS transgenic trees grown in the greenhouse, and four independent lines were analyzed to ensure reliability of the results. Consistent GUS staining results were recorded in representative transgenic lines, and wild-type plants were used as a negative control. Various tissues were incubated overnight at 37 °C in a GUS staining solution as previously described [68]. After the GUS signal was generated, chlorophyll was removed from the samples several times with 75% (*v*/*v*) ethanol. The images of the hand cross-sections of poplar leaf vein and the petiole were taken with a model SZX7 stereomicroscope (Olympus, Tokyo, Japan), and other tissues were taken by BX43 laboratory microscope (Olympus, Tokyo, Japan).

### 4.8. Subcellular Localization

By co-transfecting the *A. tumefaciens* strains containing 35S::*PtTLP6-GFP* vector with the *A. tumefaciens* strains containing mt-rk-mCherry vector for mitochondria localization or px-rk-mCherry vector for peroxisome localization [34,35,36] into tobacco leaves using agrobacterium tumefaciens-mediated transformation method mentioned above [67]. After 48 h of dark culture, the treated *Nicotiana benthamiana* leaves were collected and cut into little squares, avoiding the veins, to observe fluorescence under a confocal laser scanning microscope (LSM 800, Zeiss, Oberkochen, Germany), under a 488 nm excitation laser, which is the GFP specific excitation laser, showing the localization of *PtTLP6-GFP*, and under a 610 nm excitation laser, which is the mCherry specific excitation laser, showing the localization of mitochondria or peroxisomes. The specific localization of *PtTLP6-GFP* can be determined by merging the pictures under these two laser channels.

### 4.9. Protein Extraction and Western Blot Analysis

Plant materials were ground rapidly in liquid nitrogen and homogenized in the protein extraction buffer (5 mM DTT, 50 mM Tris-HCl, 2% SDS and 200 mM NaCl, pH 8.0) on ice for 1 h and then boiled for 10 min; after centrifugation at 14,000 rpm for 10 min, the supernatants (protein extracts) were collected for SDS-PAGE analysis. Total proteins were separated on a 10% SDS-PAGE gel and transferred into a PVDF membrane. The membrane was blocked in blocking solution overnight at 4 °C. Detailed experimental procedures were carried out as described previously [69]. The signals were captured by ECL Western Blotting Substrate (Thermo Fisher Scientific, Waltham, MA, USA) by exposure to X-ray films.

### 4.10. Histochemical Determination of H_2_O_2_

Leaves of the *PtTLP6-OE Arabidopsis* were treated with 3,3′-diaminobiline (DAB) solution: 1 mg/mL DAB, pH 3.8 [70]. In the presence of peroxidase, DAB can react with H_2_O_2_ to form a dark-brown polymerization product. After removing chlorophyll in leaves by boiling with a glycerol/acetic acid/ethanol (1:1:3, *v*/*v*/*v*) solution; the dark-brown product could be imaged with a model SZX7 stereo microscope (Olympus, Tokyo, Japan). Three biological experiment replicates and three technical experiment replicates were performed to ensure the reliability of the results.

### 4.11. Trypan Blue Staining

Trypan blue staining assay was performed as previously described [71]. In brief, leaves were immersed in trypan blue staining solution, containing 10 g phenol, 20 mL H_2_O, 10 mL lactic acid, 10 mL glycerol, and 10 mg trypan blue, boiled for 1–2 min, and then cooled down at room temperature for at least 1 h. Stained leaves were immersed in the chloral hydrate solution (50 g chloral hydrate dissolved in 20 mL water) and finally equilibrated with 70% glycerol. Three biological experiments and three technical experiment replicates were performed to ensure the reliability of the results.

### 4.12. Statistical Analysis

All data analyses and statistical tests were conducted by SPSS version 24.0. Values are displayed as the means ± standard deviation (SD), and the number of asterisks indicates statistical significance at different levels (* *p* < 0.05 and ** *p* < 0.01).

## Data Availability

Data is contained within the article and Supplementary Materials.

## Supplementary Materials

The following supporting information can be downloaded at: https://www.mdpi.com/article/10.3390/ijms25115990/s1.
