# Supplementary material for: Genome-Wide Identification of TLP Gene Family in Populus trichocarpa and Functional Characterization of PtTLP6, Preferentially Expressed in Phloem
Source: Int J Mol Sci. 2024 May 30;25(11):5990. doi: 10.3390/ijms25115990 (PMC11173255; doi:10.3390/ijms25115990)
Supplement: Supplementary file 1 [file ijms-25-05990-s001.zip › supplemental figures.pdf]

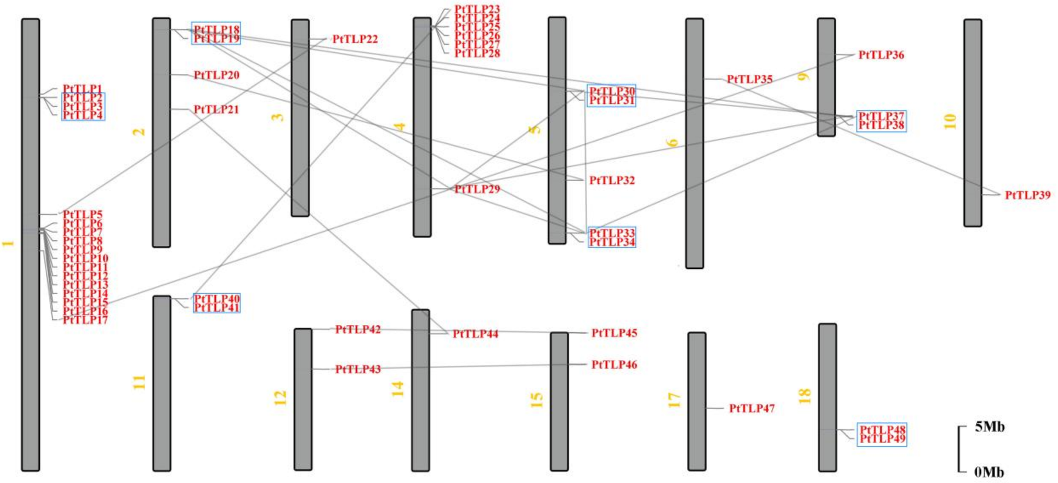

Figure S1. Gene duplications of 49 *PtTLP* genes on 14 chromosomes of P. trichocarpa. The segmental duplication genes were connected by solid gray lines. The tandem gene pairs were outlined with small blue boxes. The scale bar represents 5.0 Mb chromosome distance.

A

```
PtTLP18         ---MDRI-FTSSALHITLILLTIC---KG-LSGATFTFINRCGYTVWPGILSNAGSTPLE  52
PtTLP29         MGYYSCC-FPTLISSFVLLLLVSY---KGCVLAATFTFVNKCEHTVWPGILANAGSPRLD  56
PtTLP30         ---MDLLSFSQNHPHYCAAFLSLLFIFEG-VSGAKFTFVNKCDFSVWPGIL---GSPELD  53
PtTLP33         ---MSQL---TLLIPLSSFFISHFFI-QA-VVSTTFTLTNKCDYTVWPGSLSNADAPALS  52
PtTLP37         ---MDHL-FLSLTL-FALIPLSSF---SG-VEPITFKFINKCRHTIWPGLLSGAGTSQLP  51
                    .               :      .. :   .*.: *.* .::*** *   .:. *

PtTLP18         STGFELPQGGSRSFQAPPNWSGRFWGRTGCTFDPNTGQGTCLTGDCSSNQIECNGKNANP  112
PtTLP29         STGFELQQDSSRSFIAPTGWSGRFWARTGCNFD-ESGAGLCSTGDCGSGQVECNGFGAAP  115
PtTLP30         STGFELRKGSSRSFQAPTGWSGRFWGRTGCNFD-SSGHGSCVTADCGSGQVECNGAGATP  112
PtTLP33         TTGFALQNGESKTITAPASWGGRFWGRTYCSQD-STGKFSCVTGDCGSGKLECSGTGAAP  111
PtTLP37         TTGFYLNSGKSRTVKIPRSWSGRLWGRTFCSQD-RTGKFSCLTGDCGSGKVECLGSGAQP  110
                :*** * .. *.:.  * .*.**:*.** *. *  :*   * *.**.*.::** * .* *

PtTLP18         PATLAEFTV-GSGVLDFYDVSLVDGYNLPMIVEPNGGSGS-CLSTGCVTDMNQQCPTELR  170
PtTLP29         PATLAEFTL-GSGGQDFYDVSLVDGYNLPMVVEGSGGSGM-CASTGCISDLNIQCPQELR  173
PtTLP30         PATLAEFTL-GSGSQDFYDVSLVDGYNLPMIVEVSGGSGE-CASTGCVTDLNRKCPTELR  170
PtTLP33         PATLAEFKLDGYGGMDYFDVSLVDGYNLPLLVVPQGGSGQNCTSTGCVVDLNDSCPSELK  171
PtTLP37         PATLAEFTLNGADGLDFYDVSLVDGYNLPMLVIPKKVTRGGCGATGCLIDLNGACPKELR  170
                *******.: * .  *::***********::*  .  :   * :***: *:*  ** **.

PtTLP18         AD------SGQACKSACEAFGSPEYCCSGAYGSPDTCKPSVYSEMFKAACPRSYSYAYDD  224
PtTLP29         VG------EGDACKSACEAFGSPEYCCSGAFNTPATCKPSVYSEMFKAACPKSYSYAYDD  227
PtTLP30         TE------GGSACRSACEAFGKPEYCCSGEYNSPASCKPSMYSQVFKSACPKSYSYAYDD  224
PtTLP33         VTSTEG--ESVACKSACEAFGSPQYCCNGAYSTPDTCRPSTYSEIFKNACPRAYSYAYDD  229
PtTLP37         LSAASGGKGAVACRSACEAFGDPRFCCSEGYATPETCGPSVYSLFFKHACPRSYSYAYDD  230
                          . **.*******.* :**.  : :* :* ** ** .** ***.:*******

PtTLP18         ATSTFTCTGADYVITFCPSL-TSQKSARGNSPPASTTN--------------------  261
PtTLP29         ATSTFTCSGADYTVTFCPSS-PSQKSSSYSTPTTEGTTTSQGSGTGSGLEYTGSGSGIDS  286
PtTLP30         ATSTFTCTGADYTITFCPNF-HSLKSSSDASPQAIGGAAIGGSGTEIG------------  271
PtTLP33         KTSTFTCAAADYQITFCPSPNTSQKASQGQNTE---------------------------  262
PtTLP37         KTSTYTCAATDYDIIFCPLPYTSQKLLALRKD----------------------------  262
                 ***:**:.:** : ***    * *     .

PtTLP18         --GSETVSGDGPVGMDTSWLPNFLSGDSPNALSCSVWQFTLILS---TISCLLLSLVH--  314
PtTLP29         GAGSGTGSGGETMLADGSWLAGLAMGDSYKTASPSALQSAIMAF---TTLVLIFALLHS-  342
PtTLP30         --GTVTGSSAQEAELQSSWLASLAIGESTGTQTHHSLVLQFALV---LATSLILANSHL-  325
PtTLP33         --NTSTNSYTPLVNSTMVYEGALNQNGASPSMNSKVLGSHVIAG---IVSLTVAIWQLG-  316
PtTLP37         ------GVELPLVNKTTMY------RRSHGASSPGVVQQQFLAGAASIVTALLLFWPPLF  310
                                :        :  : .       .          :

PtTLP18         --                                                          314
PtTLP29         --                                                          342
PtTLP30         --                                                          325
PtTLP33         --                                                          316
PtTLP37         PL                                                          312
```

B

Identity / Similarity	PtTLP18	PtTLP29	PtTLP30	PtTLP33	PtTLP37
PtTLP18		59.55%	54.05%	52.20%	50.00%
PtTLP29	69.38%		62.39%	49.86%	47.78%
PtTLP30	66.07%	73.50%		49.86%	46.37%
PtTLP33	66.86%	62.74%	62.18%		53.37%
PtTLP37	60.47%	59.44%	57.82%	67.45%	

Figure S2. Sequence alignment of the reciprocal duplicate gene group *PtTLP18/29/30/33/37*. (A) The sequence alignment was carried out by MUSCLE (https://www.ebi.ac.uk/jdispatcher/msa/muscle (accessed on 30, April, 2024)). Positions which have a single, fully conserved residue were showed as '*'; ':' represents residues with particularly close properties; '.' indicates that residues that are marginally close in nature. (B) The sequence identity and similarity of *PtTLP18/29/30/33/37* with each other. The data in the upper right represented the percentage of sequence identity and the data in the lower left represented the percentage of sequence similarity.

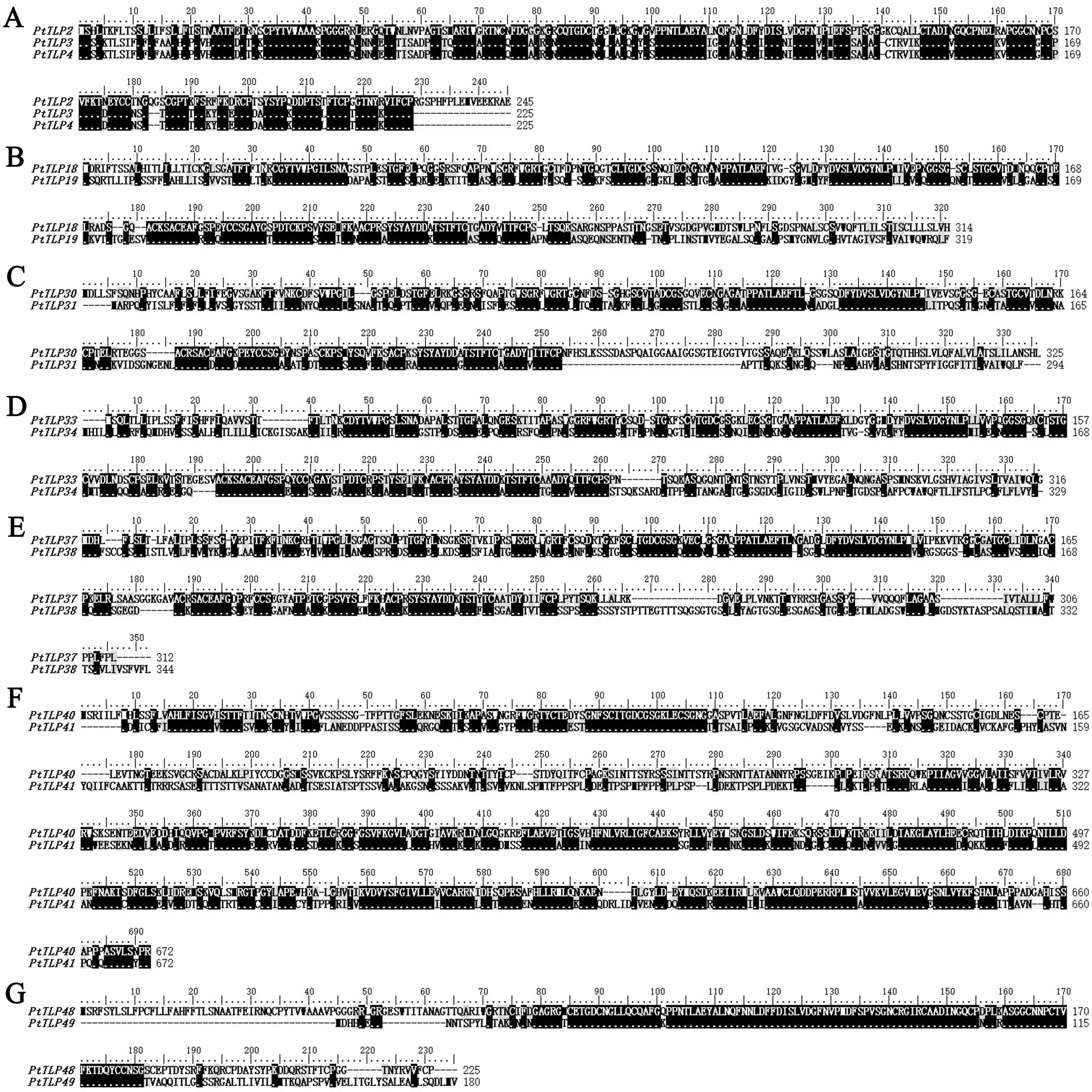

Figure S3. The sequence alignment of seven *PtTLP* tandem gene pairs. The sequence alignment was carried out by BioEdit. The background of positions which have a identity or similar residue were marked in black or gray.

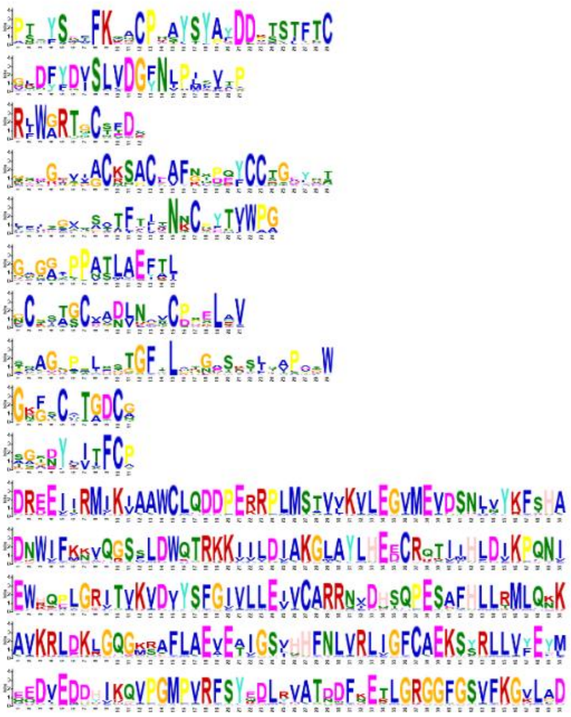

	Name	Alt. Name	Width
1.	PTQYSEFFKQACPQAYSYAYDDKTSTFTC	MEME-1	29
2.	GLDFYDVSLVDGFNLPISVTP	MEME-2	21
3.	RLWGRTGCSFDS	MEME-3	12
4.	GSDGKVIACKSACEAFNSPZYCCTGEYNT	MEME-4	29
5.	LFISGVQSATFTITNNCPYTVWPG	MEME-5	24
6.	GAGGAPPATLAEFTL	MEME-6	15
7.	GCPSTGCVADLNAVCPSELAV	MEME-7	21
8.	SNAGSPPLSSTGFTLATGASKSJSAPASW	MEME-8	29
9.	GKFSCATGDCG	MEME-9	11
10.	SGSBYLITFCP	MEME-10	11
11.	DREEIIRMIKIAAWCLQDDPERRPLMSTVVKVLEGVMEVDSNLVYKFSHA	MEME-11	50
12.	DNWIFKKVQGSSLDWQTRKKIILDIAKGLAYLHEDCRQTIIHLDIKPQNI	MEME-12	50
13.	EWHQPLGRITVKVDVYSFGIVLLEIVCARRNVDHSQPESAFHLLRMLQKK	MEME-13	50
14.	AVKRLDKLGQGKRAFLAEVEAIGSVHHFNLVRLIGFCAEKSYRLLVYEYM	MEME-14	50
15.	ZEDVEDDHIKQVPGMPVRFSYZDLRVATDDFKETLGRGGFGSVFKGVLAD	MEME-15	50

Figure S4. Fifteen conserved motif sequence logo graph of 49 *PtTLP* genes family. The relative size of the letters represents their frequency in the sequence. The height of each letter is proportional to the frequency of occurrence of the corresponding base at that position. The width of these motifs were marked on the diagram.

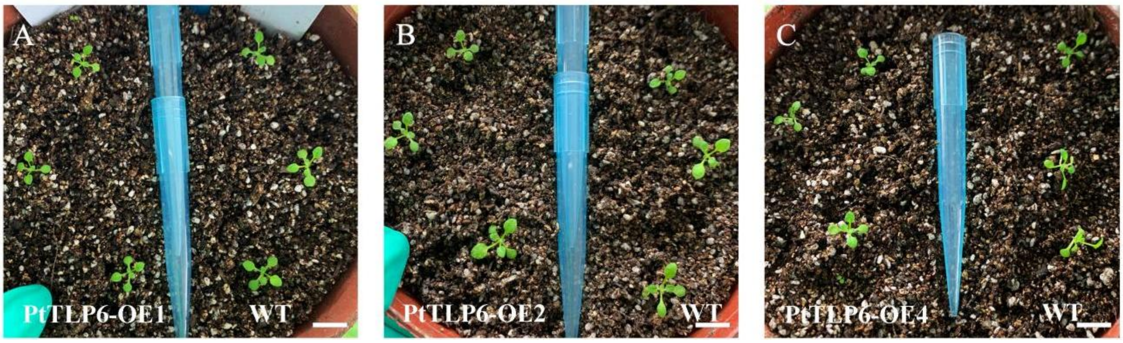

Figure S5. Phenotypes of 10-day-old *PtTLP-OE* lines and WT. The phenotypes of the 10-day-od three OE lines were similar, and none of them differed significantly from WT. Bars, 1 cm.

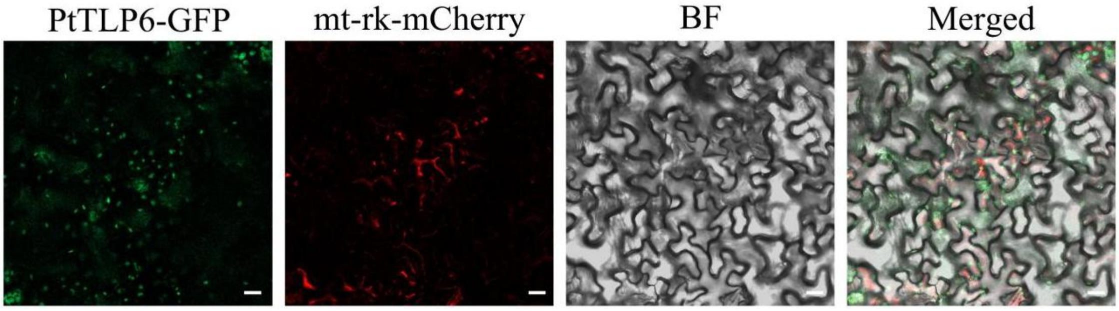

Figure S6. *PtTLP6* is not localized in mitochondria. As shown in the diagram, the *35S::PtTLP6-GFP* signal was not co-localized with the signal of mitochondria marker Mt-rk-mCherry. Bars represent 20 μm.
